# Trichosporon Urinary Tract Infections: A Hidden Menace Revealed

**DOI:** 10.30699/ijp.2025.2039595.3348

**Published:** 2025-08-15

**Authors:** Ahmad Elmimoghaddam, Mahmoud Vahidi, Reza Heidari, Mahdi Ghorbani, Peyman Aslani

**Affiliations:** 1 *Medical Biotechnology Research Center, AJA University of Medical Sciences, Tehran, Iran*; 2 *Department of Medical Laboratory Sciences, School of Allied Medical Sciences, AJA University of Medical Sciences, Tehran, Iran*; 3 *Research Center for Cancer Screening and Epidemiology, AJA University of Medical Sciences, Tehran, Iran*; 4 *Department of Parasitology & Mycology, Faculty of Medicine, AJA University of Medical Sciences, Tehran, Iran*

**Keywords:** Microbial sensitivity test, Cutaneotrichosporon, Trichosporon, Urinary Tract Infections

## Abstract

Urinary tract infections (UTIs) caused by *Trichosporon* are a significant concern for hospitalized patients and those with weakened immune systems. This narrative review study aims to provide a comprehensive overview of UTIs caused by *Trichosporon*, including its frequency, risk factors, laboratory diagnostic aspects, drug resistance, and the importance of accurate identification in clinical settings. A search of international databases was conducted to identify relevant studies, and it was found that *Trichosporon asahii*, specifically the G1 type, is the predominant causative agent of UTIs among various *Trichosporon* species. Prolonged hospitalization and immunosuppressive drug use were identified as significant risk factors for this fungal infection. Conventional methods for laboratory identification are commonly used. Still, rapid and accurate tools such as Matrix-Assisted Laser Desorption-Ionisation-Time of Flight Mass Spectrometry (MALDI-TOF MS) and DNA sequencing can improve the diagnostic process. Against all *T. asahii* isolates for which this triazole, polyene, and echinocandin were tested, voriconazole demonstrated the most potent *in vitro* activity, while amphotericin B had high MIC values and echinocandins had inherent resistance. This review provides valuable insights into the clinical significance and management of UTIs caused by *Trichosporon*.

## Introduction

A urinary tract infection (UTI) is an umbrella term used to describe infections affecting any part of the urinary tract. This includes infections of the bladder, urethra, kidneys, and other associated structures (1). There are an estimated 130 to 175 million cases of UTIs that occur globally (2). In hospital-acquired infections, the occurrence of UTIs was evaluated and found to be 12.9% in the United States, 19.6% in Europe, and 24% in developing nations (3). Urologists must not underestimate the importance of urinary tract infections (UTIs), considering their significant increase in occurrence in recent decades. The elevated frequency of UTIs, as well as the associated morbidity and mortality, signifies a critical challenge, particularly in developing countries (4). The occurrence of UTIs is strongly influenced by age and gender (5). During the year 2019, the age-standardized mortality rate (ASMR) attributable to UTIs stood at 3.13 per 100,000 individuals (6). UTIs are known to negatively affect patients' quality of life and impose a substantial clinical and economic burden. UTIs caused by bacteria and yeasts are a prevalent form of infection that is observed globally (7). UTIs caused by yeasts are predominantly instigated by *Candida* species, followed by *Trichosporon* species and other fungi may also be responsible for such infections (8–10). *Trichosporon* species are causative agents of surface infections such as white piedra, affecting hair at diverse anatomical sites (11–13). These species have also been gaining recognition as opportunistic pathogens that can result in invasive diseases (Trichosporonosis), particularly in patients with weakened immune systems (14,15). The outlook for patients suffering from invasive trichosporonosis is a matter of great concern, as various studies have reported crude mortality rates ranging from 42% to 87% (16,17). In a recent taxonomic review, 20 distinct species were identified within the genus through analysis of IGS1 rDNA sequences (18). Within the group of 20 distinct species, *Trichosporon asahii*, *T. asteroides*, *T. inkin*, *T. ovoides*, and *T. faecale *have been reported to cause infections in humans. The primary culprit responsible for invasive trichosporonosis, an emerging infectious disease and UTIs is *T. asahii* (19–21). Based on the revised taxonomic scheme introduced in 2015, *T. cutaneum*, *T. dermatis*, *T. jirovecii*, and *T. mucoides* have undergone reclassification and are now classified under the genus *Cutaneotrichosporon*. *Cutaneotrichosporon* species are frequently encountered in both human and animal sources, including clinical specimens, and certain strains have been linked to opportunistic infections (18). UTIs caused by *T. asahii* are atypical invasive infections and have been infrequently reported in medical literature. These infections are typically observed in hospitalized patients (21). In a study conducted by Sabharwal in 2010, it was observed that *T. asahii *was the predominant fungus in patients with UTIs who also had diabetes (22). The prevalence of medically significant species like *Trichosporon* spp. that are infrequently encountered has risen in recent years due to various reasons. These include the higher incidence of degenerative and cancerous diseases, greater exposure of patients to immunosuppressive drugs, chemotherapy, and broad-spectrum antibiotics, and increased use of invasive medical procedures like urinary or intravenous catheters, endoscopic forceps, and arteriovenous grafts (14,16,17,23–26). A considerable number of cases of breakthrough trichosporonosis have been frequently observed in immunocompromised patients, particularly following the use of ineffective antifungal therapies such as amphotericin B, echinocandins, and, less commonly, triazoles (16). *Trichosporon* is a newly emerging infection that has been increasingly observed in invasive forms, which is alarming. This fungus exhibits inherent resistance to conventional antifungal treatments, rendering it even more severe. Appropriate and timely diagnosis and management are vital in managing this infection effectively (15). *T. asahii* is an emerging opportunistic infection and the predominant pathogenic fungus within its genus, causing urinary tract infections in individuals with weakened immune systems (27,28). Although these cases are rarely documented in scientific literature, more information is needed to understand the risk factors, potential complications, and susceptibility of different species to antifungal drugs. 

The identification of *T. asahii* in urine culture specimens from hospitalized patients is a therapeutic challenge due to the absence of clearly defined and specific guidelines for clinical interpretation and treatment (16). Moreover, the testing for antifungal susceptibility lacks standardization, and there is an absence of interpretive epidemiological cut-off values for minimal inhibitory concentrations that can differentiate non-wild type *Trichosporon* isolates (29). Due to the lack of comprehensive research on the frequency of urinary tract infections caused by *Trichosporon*, a review of all *Trichosporons* isolated from the urine of patients around the world during the last four decades will be conducted in this study. By analyzing patient demographics, geographical location, and other contributing factors, patterns and trends can be identified, precise statistics on the prevalence of these infections can be obtained, major risk factors can be identified, and the susceptibility of various *Trichosporon* species to commonly used antifungal drugs can be evaluated based on global research findings.

## Materials and methods

For this narrative review, a thorough electronic search was performed on international databases to collect pertinent studies published before May 16, 2023. The search was conducted on reputable platforms such as PubMed, Scopus, Google Scholar, and Web of Science. Several specific search terms, such as "urinary tract infection", "urinary tract infections", "UTI", "urine", "funguria", "*Trichosporon*" and "*Cutaneotrichosporon*" were utilized in various combinations during each database search.

## Results

### Study Selection

The study collected a total of 729 articles from designated databases, which were then subjected to a deduplication process. This resulted in 492 unique articles that were considered suitable for further scrutiny and analysis. The study excluded articles written in languages other than English, as well as review papers, case reports, conference proceedings, letters, books, editorials, and notes. The main objective was to focus exclusively on original research articles for the purpose of analysis. Following a thorough examination of the titles and abstracts of the 492 remaining original papers, 46 articles were chosen to advance into the primary phase of the study.

### Study Characteristics

This narrative review analyzed 46 articles. These findings are summarized in Table 1 and cover a 33-year period from 1989 to 2022 (Table 1). Asia has been the site of 45.65% of the studies, while America accounts for 30.43%. Following these regions in terms of frequency of study are Europe and Africa. A majority of research studies (60.8%) were carried out in the period starting from 2010 and continuing until the present time.

A restricted number of studies have specifically explored UTIs attributed to *Trichosporon*. Nevertheless, this narrative review encompasses a number of original investigations that documented the identification of *Trichosporon* isolates derived from urine samples.

**Table 1:** The detailed information presented in the 46 included studies.

Row	Year of Publication	Country	No. of Positive Yeast Cultures	Frequency%	Isolates Distribution	Diagnostic Methods	Sex	MeanAge(Years)	Risk Factors	Reference [Number]
1	2022	Thailand	*	*	*T. asahii* (51)*T. inkin* (1)*Trichosporon *spp*.* (1)	MALDI-TOFDNA-Sequencing (IGS1)Conventional	NA	NA	Hospitalization (53)	(30)
2	2021	Turkey	1442	6.9%	*T. asahii* (100)	MALDI-TOF MS	M (68)F (32)	69.94	ICU (82)	(31)
3	2021	Brazil	*	*	*T. asahii* (157)	DNA-sequencing (IGS1)	NA	NA	NA	(32)
4	2021	India	*	*	*Trichosporon *spp*.* (15)	VITEK MS	NA	NA	NA	(33)
5	2020	Iran	7	14.3%	*T. asahii* (1)	DNA-sequencing (ITS)	M (1)	67	Kidney transplantation (1)	(34)
6	2019	India	11	18.2%	*Trichosporon *spp*.* (2)	Conventional	NA	NA	Burn patients (2)	(35)
7	2019	Egypt	41	4.9%	*C. mucoides* (2)	VITEK 2	NA	NA	Cancer (2)	(36)
8	2019	Mexico	51	15.7%	*T. beigelii* (8)	VITEKConventional	NA	NA	NA	(37)
9	2019	Iran	135	3%	*T. asahii* (4)	DNA-sequencing (ITS)	M (1)F (3)	41.5	Diabetes Mellitus (3)Renal Failure (1) Hospitalization (3)	(38)
10	2018	Mexico	*	*	*T. asahii* (33)	API 20CDNA-Sequencing (IGS1)Conventional	NA	NA	NA	(39)
11	2018	Taiwan	508	0.6%	*T. asahii* (3)	NA	NA	NA	NA	(40)
12	2018	Turkey	*	*	*T. asahii* (68)	MALDI-TOF MSVITEK MSDNA-sequencing (IGS1) Conventional	M (43)F (25)	65	Hospitalization (68)ICU (63%)Urology ward (14%)Nephrology ward (12%) Oncology ward (5%) Hematology clinic (4%) Neurology ward (2%)Chronic disease (68)Urinary Catheter (51)	(41)
13	2018	Spain	155	0.6%	*T. asahii* (1)	MALDI-TOFAPI 32CConventional	NA	Over 80 Years Old	Hospitalization (1) Above 80 years (1)	(42)
14	2017	Korea	14	14.3%	*T. asahii* (2)	VITEK 2 Conventional	NA	NA	NA	(43)
15	2017	Taiwan	19	5.3%	*Trichosporon *spp*.* (1)	Conventional	NA	NA	Hospitalization (1)Urinary Catheter (1)	(44)
16	2016	Brazil	*	*	*T. asahii* (9)	DNA-sequencing (IGS1)	NA	NA	NA	(45)
17	2015	Brazil	54	3.7%	*Trichosporon *spp*.* (2)	VITEK 2Conventional	NA	NA	NA	(46)
18	2015	Egypt	23	8.7%	*T. beigelii* (2)	Conventional	NA	25-85years old	Urinary Catheter (2)Bladder cancer (2)	(47)
19	2014	India	123	3.3%	*Trichosporon *spp*.* (4)	Conventional	NA	NA	NA	(48)
20	2014	Brazil	*	*	*T. asahii* (20)	DNA-sequencing (IGS1)	NA	NA	NA	(49)
21	2014	Korea	159	5.0%	*Trichosporon *spp*.* (8)	Conventional	NA	Over 18 Years Old	Urinary Catheter (8)	(50)
22	2014	Argentina	*	*	*T. asahii* (9) *Trichosporon *spp*.* (1)	DNA-sequencing (IGS1, ITS, D1/D2)	NA	NA	Hospitalization (10)	(51)
23	2014	Spain	*	*	*T. asahii* (32)	VITEK 2 DNA-sequencing (IGS, ITS)	M (26)F (6)	85	Urine drainage bag (32)Hospitalization (32) Antibiotic therapy (32) Pneumonia (12) Sepsis (8) Kidney diseases (6) Cancer (1) ICU (1)	(52)
24	2014	India	337	2.1%	*Trichosporon *spp*.* (7)	VITEK 2	NA	NA	Chronic liver disease (7)Urinary Catheter (7)	(53)
25	2012	China	*	*	*T. asahii* (23)	VITEK 2DNA-sequencing (IGS1, ITS)	M (17)F (6)	80	ICU (23)Systemic antibiotics (14) Urinary Catheter (14) Diabetes mellitus (8)High blood pressure (7) Heart failure (5)Chronic Diseases (2)	(54)
26	2012	Taiwan	*	*	*T. asahii* (8) *T. montevideense *(2)*C. dermatis *(1)*C. cutaneum* (1)*T. japonicum *(1)	DNA-sequencing (ITS, D1/D2)API 32C VITEK	NA	NA	NA	(55)
27	2011	Korea	93	1.1%	*T. asahii* (1)	VITEK 2	NA	NA	Burn patients (1) Urinary Catheter (1)	(56)
28	2011	Brazil	27	3.7%	*T. beigelii* (1)	VITEK 2	NA	NA	Urinary Catheter (1)Hospitalization (1)	(57)
29	2010	Turkey	*	*	*T. asahii* (23)	API 20CRep-PCR	M (10)F (5)	44	Hospitalization (15) Urinary Catheter (15)	(58)
30	2009	Poland	*	*	*T. asahii* (22)	ATB Expression	NA	NA	Kidney transplantation (21) Simultaneous pancreas Kidney transplantation (1)	(59)
31	2009	Taiwan	*	*	*T. asahii* (9)*C. dermatis *(1)*T. montevideense *(1)	DNA-sequencing (IGS1)API 32CConventional	NA	NA	Hospitalization (11)	(25)
32	2009	Qatar	*	*	*T. asahii* (6) *T. faecale *(1)	DNA-sequence (LSU, ITS) VITEK 2API 32CConventional	M (5)F (2)	47.1	Pyuria (7)Hematuria (1)	(60)
33	2009	Poland	12	8.3%	*Trichosporon *spp*.* (1)	API 32C Conventional	NA	NA	hematological malignancies (1)	(61)
34	2009	Turkey	28	3.6%	*Trichosporon *spp*.* (1)	Conventional	NA	NA	ICU (1)	(62)
35	2008	Brazil	*	*	*T. asahii* (8)	API 20CConventional	NA	NA	ICU (8)	(63)
36	2008	Brazil	*	*	*T. asahii* (3)	DNA-sequencing (IGS1, ITS)	NA	NA	Hospitalization (3)	(64)
37	2007	India	145	5.5%	*T. beigelii* (8)	Conventional	NA	NA	Hospitalization (8)	(65)
38	2007	Brazil	100	3%	*T. asahii* (3)	Conventional	NA	0-7years old	Hospitalization (3)Candiduria (3)	(66)
39	2005	Kuwait	*	*	*T. asahii* (19)	VITEK 2DNA-sequencing (ITS)	NA	NA	Cancer (4)Kidney failure (1)Kidney transplantation (1)Severe burns (1)Hemi-colectomy (1)Low birth weight (1)	(67)
40	2005	Spain and Argentina	*	*	*T. asahii* (2) *C. jirovecii *(1)	DNA-sequencing (IGS1, ITS)Conventional	NA	NA	Hospitalization (3)	(68)
41	2000	Canada	*	*	*T. beigelii* (11)	Conventional	M (8)F (3)	42	Immunosuppressive drugs (11) Kidney transplantation (11)broad-spectrum antibiotics (6) Urinary Catheter (5)	(69)
42	1993	Japan	39	10.2%	*Trichosporon *spp*.* (4)	Conventional	NA	NA	NA	(70)
43	1992	India	9	11.1%	*C. cutaneum* (1)	Conventional	NA	NA	Acute leukemia patients (1)	(71)
44	1992	Saudi Arabia	302	0.3%	*T. beigelii* (1)	API 20CConventional	NA	NA	Hospitalization (1)	(72)
45	1989	USA	*	*	*T. beigelii* (15)	VITEK	M (11)F (4)	18-85years old	Hospitalization (15) Urinary Catheter (15)	(73)
46	1966	USA	179	1.1%	*C. cutaneum* (2)	Conventional	F (2)	NA	NA	(74)

* *Trichosporon *and* cutaneotrichosporon* were the sole microorganisms identified in the urine samples within the statistical population of these studies.

### Risk Factors

Individuals afflicted with various types of cancer, receiving chemotherapy treatment, displaying neutropenia, experiencing severe burns or cystic fibrosis, or diagnosed with advanced kidney failure, as well as those with compromised immune systems, are more susceptible to the development of severe trichosporonosis (75–77). In a 2015 study, it was discovered that UTIs caused by *Trichosporon* had an occurrence rate of 6% within the intensive care unit (ICU) over two years. Furthermore, these infections were found to be associated with a significant mortality rate of 20% (9). Based on the findings of the studies, hospitalization, utilization of urinary catheters, receipt of organ transplants, and administration of antibiotic therapy are identified as the prevalent risk factors for urinary trichosporonosis.

### Diagnosis

Yeast-like colonies are obtained through culturing on sabouraud dextrose agar. These colonies exhibit a cream-colored appearance, which may eventually transition to a yellowish-grey hue. The colonies possess significant wrinkling, with the center appearing heaped and folded. Additionally, they tend to adhere to and cause cracking of the agar surface. The process of diagnosing the condition is complicated as it involves identifying a yeast-like organism in a clinical sample (78). The use of direct examination is not always helpful for making a conclusive diagnosis since it does not commonly exhibit arthroconidia and has histological similarities with Candida. Nevertheless, this organism can be differentiated from *Candida* through its thinner hyphae and pseudohyphae and its slight staining with Gomori methenamine silver (GMS) stain (79). The intergenic spacer 1 (IGS1) region of the gene is an essential factor in the precise and distinct identification of different *Trichosporon* species (80,81). During a research investigation analyzing 45 clinical isolates and three reference isolates of *Trichosporon* species, assessments that focused on both the internal transcribed spacer (ITS) region and the intergenic spacer 1 (IGS1) region successfully distinguished all 48 isolates (80). The molecular identification of *Trichosporon* species in clinical samples obtained from Indian patients has been accomplished using the IGS1 region (82). In a reverse line blot (RLB) hybridization and rolling circle amplification (RCA)-based assay, accurate identification of *Trichosporon* species was achieved utilizing species-specific probes directed towards the ITS region and the D1/D2 domain. This technique exhibited 100% specificity in identifying the species when compared to DNA sequencing results from the ITS region, D1/D2 domain of the 28S rRNA gene, and IGS1 region (81). Successful management of invasive trichosporonosis infections is contingent on the diagnostic method's sensitivity and timeliness.

### Identification Method and Species Distribution

After conducting a statistical analysis of several studies, it has been observed that 170 cases out of 4013 instances of yeast urinary tract infections are linked with diverse *Trichosporon* species. These species have been identified to be responsible for causing such infections, thus indicating the high occurrence and significance of UTIs caused by *Trichosporon*-related strains. Because there is a scarcity of literature examining urinary tract infections caused by *Trichosporon*, no temporal boundaries were imposed during the search process. Nevertheless, certain investigations focused solely on candiduria or other microbial urinary infections, which meant that *Trichosporon* was only identified at the level of genus and not down to the specific species. Furthermore, some studies had limited access to precise identification tools, leading them to limit their identification of *Trichosporon* to the genus level. Over time, multiple methods have been utilized to identify *Trichosporon* at the genus or species level. In the past, identification techniques were primarily limited to morphology, microscopic and macroscopic examination, and different sugar fermentation tests. Over time, the progress in microbial identification techniques used in research and medical labs has led to the implementation of advanced methods like VITEK 2, MALDI-TOF MS, and DNA sequencing for better identification of various *Trichosporon* species. These techniques have greatly improved the accuracy of identification. Having analyzed and consolidated the statistical data reported in multiple studies, our findings indicate that *T. asahii* is the predominant type of *Trichosporon* detected from urine, with *T.* *beigelii*, *Cutaneotrichosporon cutaneum *(formerly named* T. cutaneum*), and *C. mucoides *(formerly named* T. mucoides*) following in descending order (Chart 1) (31,34-38,40,42-44,46-48,50,53,56,57,61,62,65,66,70 -72,74). 

**Fig 1:**
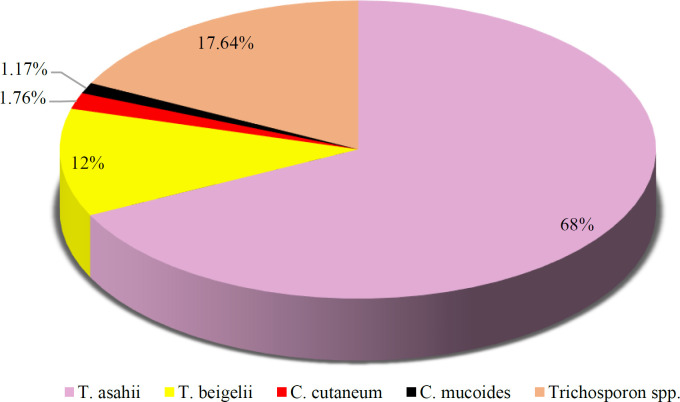
The variability of distinct Trichosporon/Cutaneotrichosporon species obtained from urine across 25 investigations.

### Genotype of T. asahii

To identify the genotype of *T. asahii* isolates obtained from urine, a total of nine studies were conducted. The results revealed that more than 69% of the *T. asahii* isolates belonged to genotype type I (G1) while genotypes G3 and G7 were found to be the subsequent most common genotypes (Chart 2) (30,32,39,41,45,51,52,54,64).

### Antifungal Susceptibility Patterns:

*In vitro* antifungal susceptibility testing (AFST) was performed on *T. asahii* isolates obtained from urine during studies published before May 16, 2023. The isolates were tested for susceptibility to amphotericin B, 5-flucytosine, 6 triazole, and 3 echinocandin using the broth dilution method according to Clinical and Laboratory Standards Institute (CLSI) and European Committee on Antimicrobial Susceptibility Testing (EUCAST) guidelines, Epsilometer Test (E-test), and the VITEK 2 system. The results are summarized in Table 2 (20,30,31,45,49,52,58,63,64). 

**Fig 2:**
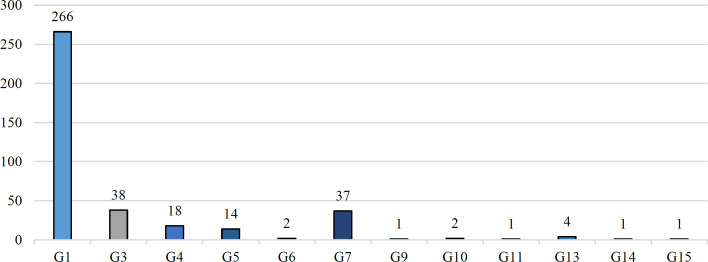
The diversity of distinct T. asahii genotypes across a given population.

**Table 2:** The summary of all data reporting antifungal susceptibility patterns of T. asahii isolated from urine during studies published before May 16, 2023 (studies without raw data of MIC are not included).

MethodIncubation time	No. of isolates with available data	Antifungal agent	(MIC) values (μg/mL)		
			MIC range	MIC_50_	MIC_90_
CLSI M2748-h	345	AMB	0.062- ≥ 16	2	4
	345	FCZ	0.25- ≥ 64	2	8
	328	VCZ	≤ 0.015 -2	0.062	0.125
	257	PSZ	0.015-2	0.25	1
	137	ITZ	0.015-8	0.25	0.5
	100	ISZ	≤ 0.008-2	0.12	0.25
	100	MYC	>8	>8	>8
	8	5FC	16-32	16	32
E-test48-h	55	AMB	0.062- ≥ 32	2	8
	55	FCZ	0.25-64	4	16
	55	ITZ	0.032-32	0.5	4
	32	VCZ	0.008-0.75	0.062	0.125
	32	CAS	≥16	≥16	≥16
	32	5FC	≥32	≥32	≥32
EUCAST EDef 7.148-h	35	AMB	0.5- 8	1	2
	35	FCZ	2-32	8	8
	35	ITZ	0.125-1	0.5	1
	35	VCZ	0.125-1	0.25	1
	35	CAS	≥16	≥16	≥16
	35	5FC	2-64	8	16
	3	PSZ	0.125-0.25-0.5	*	*
	3	MYC	16	*	*
	3	ANF	16	*	*
	3	RAV	0.125-0.5-4	*	*
VITEK 2 system	32	AMB	≤0.25-2	0.5	1.0
	32	FCZ	2.0-16.0	2.0	8.0
	32	VCZ	≤0.12-1.0	0.1	0.3
	32	CAS	≥4.0	≥4.0	≥4.0
	32	5FC	≤1.0-8.0	4.0	8.0

MIC: Minimum Inhibitory Concentration, AMB: Amphotericin B, FCZ: Fluconazole, VCZ: Voriconazole, PSZ: Posaconazole, ITZ: Itraconazole, ISZ: Isavuconazole, MYC: Micafungin, 5FC: 5-Fluorocytosine CAS: Caspofungin, ANF: Anidulafungin, RAV: Ravuconazole

Typically, *Trichosporon *infections are treated with triazole antifungal agents and amphotericin B (16). Based on growth evaluation conducted at 48 hours, it was found that the majority of strains had a MIC90 value equal to or greater than 1 µg/mL for amphotericin B. Previous investigations have indicated that amphotericin B demonstrates insufficient fungicidal activity and restricted *in vivo* efficacy, with evidence of *in vitro* resistance (83). *In vitro* studies comparing the MIC values of various azole antifungal agents, such as voriconazole, itraconazole, posaconazole, isavuconazole, ravuconazole and fluconazole, were conducted. Results indicated that triazole antifungal agents demonstrated similar and low MIC values at 48 hours, whereas fluconazole displayed higher MIC values compared to other azole agents. Among the triazole drugs, voriconazole showed the best *in vitro* activity against clinical isolates of *T. asahii* and is considered as the first-line therapy, particularly for hematological patients. The MIC results revealed that all the strains tested had MIC values exceeding 4 mg/L for the echinocandins drug. Furthermore, *Trichosporon *species showed innate resistance to echinocandins and were found to be unaffected by this class of drugs (17). 5-Flucytosine, a drug commonly used to treat fungal infections, is ineffective against *Trichosporon*. Nevertheless, certain studies indicate that administering a combination of 5-flucytosine and amphotericin B can lead to favorable outcomes in the treatment of trichosporonosis (84). The findings propose that the concomitant administration of echinocandin and amphotericin B may exhibit synergistic antifungal properties (85). Previous studies have indicated that the broth dilution method and the VITEK 2 system reveal comparable results for the susceptibility testing of amphotericin B, 5-flucytosine, fluconazole, voriconazole, and caspofungin with no discrepancies exceeding a two-dilution difference in MIC50 and MIC90 (86). The E-test is a feasible method for routine laboratory use; however, its results have been found to deviate from previous findings particularly in the case of 5-flucytosine and itraconazole, which appear to be anomalous. Lemes *et al*. have also reported significant inconsistencies between the E-test and CLSI methodologies regarding 5-flucytosine and itraconazole (87). 

## Conclusion

*Trichosporon *species infections, primarily arising from endogenous microbial populations, present a heightened threat to individuals with weakened immune systems or those confined to ICUs. This increased risk can be attributed to factors such as microbial translocation across the gastrointestinal mucosa and the presence of indwelling catheters.

Various factors, including patient age, length of hospital stay, pre-existing medical conditions, invasive medical procedures, immune status, and other variables, play a role in determining the occurrence and severity of invasive fungal infections (IFIs) such as Trichosporonosis.

The traditional methods used to identify fungi based on their physiological and morphological characteristics are not only time-consuming but also often inadequate. However, the introduction of molecular techniques such as polymerase chain reaction (PCR) with species-specific primers has revolutionized fungal infection diagnosis by providing a simpler, more specific, and faster approach. These molecular methods offer high sensitivity and specificity, enabling the differentiation of closely related species with precision and accuracy.

Due to a scarcity of studies concurrently investigating the drug sensitivity and genotypic frequency of *Trichosporon* isolates derived from urine specimens, our ability to explore the correlation between drug sensitivity and genotype was hindered. While certain studies solely examined the drug sensitivity of *Trichosporon* isolates acquired from urine, and other studies focused on determining the prevalence of diverse genotypes of *T. asahii* obtained from similar urine samples, there exists a notable dearth of research encompassing both aspects together. Consequently, we were unable to elucidate the association between drug sensitivity and genotype in this context.

Accurate identification of *Trichosporon* species is crucial to administer appropriate and effective treatment. This is because different species of *Trichosporon* exhibit varying levels of susceptibility to antifungal medications. Additionally, timely diagnosis and prompt initiation of treatment are of utmost importance for patients affected by trichosporonosis. Based on the existing data, it appears that the sensitivity of *Trichosporon* strains obtained from urine samples towards antifungal agents is subject to variation, which can be attributed to both the species and the type of drug utilized in the analysis. However, additional investigations are warranted to comprehensively elucidate the drug susceptibility patterns of *Trichosporon* strains isolated from urinary sources. Furthermore, it is worth noting that *T. asahii*, a specific species within the *Trichosporon *genus, demonstrates high resistance to the antifungal drug amphotericin B.

The isolation of various *Trichosporon *species from urine, specifically in immunocompromised patients and ICU admissions, necessitates comprehensive research efforts. These studies aim to achieve multiple objectives, including investigating the prevalence of *Trichosporon *infections in urine samples, employing molecular and sequence-based methods for identifying *Trichosporon* species, gathering accurate epidemiological data, and performing antifungal susceptibility testing for commonly used antifungal drugs. Such efforts can provide a clear, precise, and broader understanding of the management and treatment of this infection.

## Data Availability

There is no additional data separate from available in cited references.
